# Optical Fiber Sensor for Monitoring the Evaporation of Ethanol–Water Mixtures

**DOI:** 10.3390/s22155498

**Published:** 2022-07-23

**Authors:** Diana Pereira, Jörg Bierlich, Jens Kobelke, Vanda Pereira, Marta S. Ferreira

**Affiliations:** 1 i3N & Department of Physics, University of Aveiro, Campus Universitario de Santiago, 3810-193 Aveiro, Portugal; dsap@ua.pt (D.P.); vanda.pereira@staff.uma.pt (V.P.); 2 Leibniz Institute of Photonic Technology IPHT, Albert-Einstein-Str. 9, 07745 Jena, Germany; joerg.bierlich@leibniz-ipht.de (J.B.); jens.kobelke@leibniz-ipht.de (J.K.); 3 ISOPlexis—Sustainable Agriculture and Food Technology Center, University of Madeira, Campus da Penteada, 9020-105 Funchal, Portugal

**Keywords:** hollow square-core fiber, cladding modal interference, Mach–Zehnder interference, binary mixture, ethanol concentration, evaporation

## Abstract

An inline optical fiber sensor is proposed to monitor in real time the evaporation process of ethanol–water binary mixtures. The sensor presents two interferometers, a cladding modal interferometer (CMI) and a Mach–Zehnder interferometer (MZI). The CMI is used to acquire the variations in the external medium refractive index, presenting a maximum sensitivity of 387 nm/RIU, and to attain the variation in the sample concentration profile, while the MZI monitors temperature fluctuations. For comparison purposes, an image analysis is also conducted to obtain the droplet profile. The sensor proposed in this work is a promising alternative in applications where a rigorous measurement of volatile organic compound concentrations is required, and in the study of chemical and physical properties related to the evaporation process.

## 1. Introduction

The monitoring of the evaporation process of liquid species, along with the understanding of their physical and chemical properties, is a matter of deep study. Several works have been conducted to better understand this phenomenon [1,2,3]. Some of the techniques employed nowadays are based on image processing, where the analysis of a droplet profile allows us to ascertain some properties of the evaporation [4,5,6], or based on other techniques, like infrared spectroscopy [7], thermography [8], gas chromatography [9], and ion flow tube mass spectrometry [10]. An alternative method arises from the use of optical fiber sensors; this can present a higher resolution, has fast response times, and does not suffer from drift [11].

The role that volatile organic compounds (VOCs) play is of extreme importance, their use being a recursive one. Their importance is dictated not only by demand, but also by the danger they may present. These compounds are extensively used in industrial processes, in household products, and even in the food and beverages industry [11,12]. However, they can be very dangerous and a threatening element, not only to the environment [13] but also to human health, leading to severe adverse effects [11]. Thus, their detection has become an important issue and a matter of concern. Ethanol is a well-known VOC that is extensively used in several fields, among which the alcoholic beverage industry and fuel industry are highlighted, and also in the chemical and pharmaceutical sectors [14]. However, despite its numerous applications, ethanol also presents several risks to health, causing headaches, eye irritation, and difficulty breathing, among other issues [15].

Different configurations have been proposed in the literature to monitor VOCs using optical fiber sensors. For instance, Paixão et al. [16] reported the use of a suspended-core fiber sensor to detect gaseous ethanol. The sensor was able to detect different concentrations of ethanol, presenting a sensitivity of 3.9 pm/wt.%. Furthermore, Zheng et al. [17] proposed a sensor based on a core-removed D-shaped fiber to monitor in real time the evaporation of a chloroform and alcohol mixture. The device achieved a sensitivity of 10,243 nm/RIU in the range of 1.430–1.444. A long-period grating coated with a mesoporous film was proposed by Hromadka et al. to detect chloroform, benzene, toluene, and acetone vapors [18]. An optical fiber-tip sensor based on localized surface plasmon resonance was reported by He et al. for the monitoring of different VOCs. The sensor was produced by coating a multimode optical fiber tip with gold nanoparticles and subsequent functionalization with a metal–organic framework [19].

A different approach was followed by Preter et al., where the evaporation of a droplet located at the fiber tip [20] or in an in-fiber micro-cell [21] was monitored by means of optical power variations. Tip sensors based on microstructured optical fibers have also been employed to monitor the evaporation of fluids [22,23]. Many other sensing devices have been proposed to detect and monitor VOCs, and thorough reviews can be found in [11,14,15].

In this work, the use of an optical fiber sensor based on a hollow square-core fiber (HSCF) to monitor the evaporation of ethanol–water mixtures is proposed. The sensor presents two different interferometers: one that is able to detect the evaporation process by measuring real-time variations in the binary sample’s refractive index, and another that can monitor temperature fluctuations in the sample. These allow us to retrieve important information regarding the variation in the mixture concentration and, thus, the evaporation process; combined with droplet image analysis, the sensor reveals great promise in future applications.

## 2. Principle of Operation

**Figure 1:**
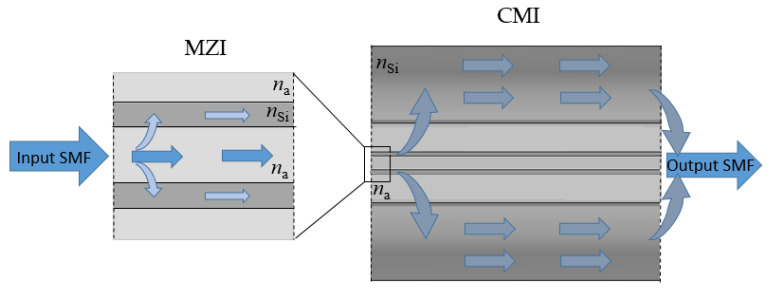
Scheme of the optical paths in the HSCF that originate the MZI and CMI, where *n*_a_ and *n*_Si_ are the refractive indices of air and silica, respectively.

The hollow square-core fiber (HSCF) is an antiresonant fiber whose light guidance relies on the antiresonance reflection within the core [24]. However, when this fiber is spliced between two single-mode fibers (SMF), other paths can be generated, giving rise to a Mach–Zehnder interferometer (MZI) and a cladding modal interferometer (CMI) [25], as depicted in Figure 1. When the light that comes from the input SMF reaches the interface of the HSCF, it will be coupled to its hollow core, enhancing multiple core modes, and will also be coupled to the silica strands that surround the core. When the output SMF is reached, the light from these two media will recombine and consequently interfere, giving rise to the MZI. On the other hand, as light propagates in the silica strands, it will tend to escape to the outer cladding, hence exciting cladding modes. Those cladding modes, which present different propagating constants, will interfere as they propagate in the HSCF. The signal is then recoupled into the output SMF, originating the CMI.

## 3. Materials and Methods

### 3.1. HSCF Sensor Fabrication

**Figure 2:**
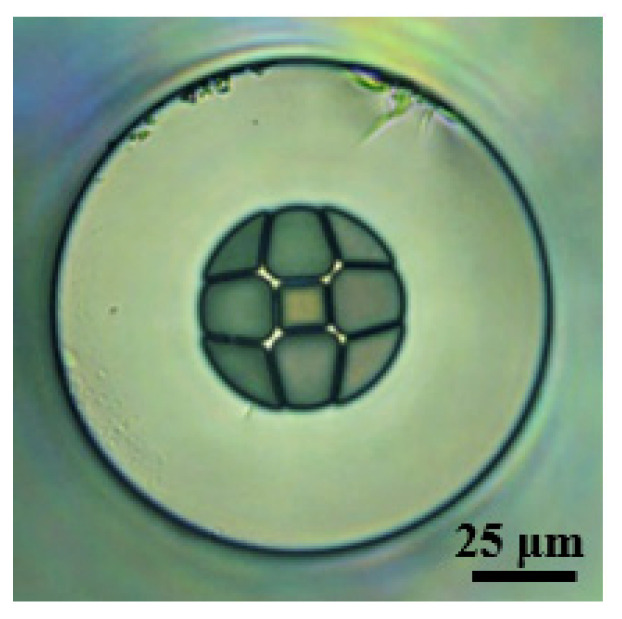
Microscopic picture of the HSCF cross section.

The HSCF used in this work, the cross section of which is shown in Figure 2, presents a square-shaped core surrounded by four identical air petal-shaped structures intercalated with four interstices. The side size of the core is 11 µm, and it is surrounded by silica strands of ~1.7 µm of thickness. The silica cladding has a width of 36.5 µm, and the fiber outer diameter matches the 125 µm of the SMF.

The layout of the sensor used in this work was based on an inline transmission configuration where a segment of HSCF with a length of 3 mm was spliced between two segments of SMF. The splicing process was executed with a Fujikura 40S splicer, which was operated in the manual mode, allowing the alignment between fibers to be personalized by the user. The fusion parameters were set as 10 arb. units (power) and 500 ms (duration). Further details can be found in [24].

### 3.2. Experimental Setup and Spectral Characterization

**Figure 3:**
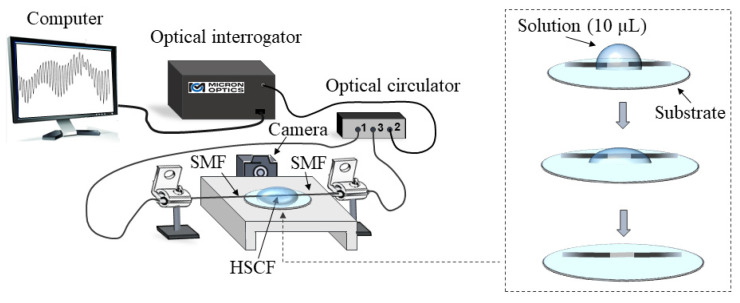
Scheme of the experimental setup.

Figure 3 displays the experimental setup used to monitor the evaporation. The sensor, placed on top of a ~10 mm diameter Teflon substrate, was interrogated in a transmission configuration. A 10 µL droplet of the solution was placed above the sensing head, using a micropipette. Notice that this volume was carefully chosen so that the droplet of the solution would be enough to cover the whole sensing head, and so that the duration of the evaporation process would not be too long. To monitor the evaporation process, the sensor spectral response was acquired over time, with an acquisition rate of 10 s, using an optical interrogator (Micron Optics SM125, Micron Optics, Inc., Atlanta, GA, USA) with a resolution of 5 pm. Simultaneously, a digital camera was used to acquire the droplet profile. This procedure was performed continuously until the droplet was completely evaporated.

**Figure 4:**
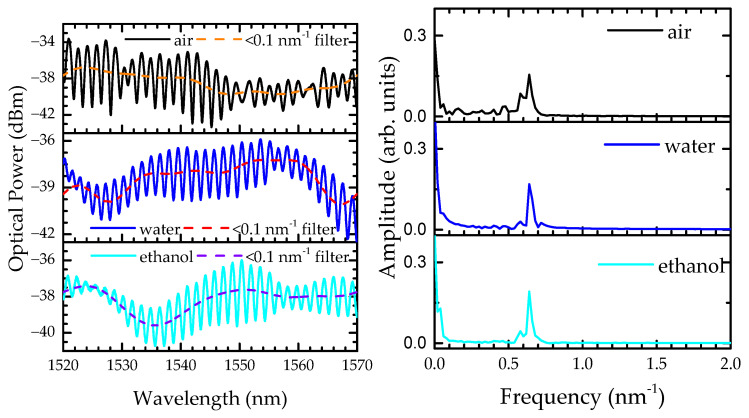
(**left**) Transmission spectra of the 3 mm long sensor when surrounded by air, water, and ethanol. The dashed lines represent the lowpass filter used to monitor the CMI. (**right**) Respective FFT graphs of the sensor when surrounded by air, water, and ethanol.

Figure 4 presents the spectral responses of the sensor (left) with the respective fast Fourier transform (FFT) graphs (right) when surrounded by air, water, and ethanol. As previously reported [25], the sensor response in liquid media is modulated by two interferometric phenomena, the CMI (corresponding to the low frequencies located at ~0.1 nm^−1^) and the MZI (corresponding to the high frequencies located at ~0.6 nm^−1^). The spectral response of the sensor changes significantly when there is a transition from air medium to liquid media, which is also observable in the FFT (air), where more peaks, with a higher amplitude, appear in the low frequencies. This is due to the larger number of modes propagating in the cladding region when the sensor is surrounded by air. When the sensor is submerged in liquid, due to the increase in the external medium refractive index, some of the modes will fade away. This behavior is illustrated in the FFT graphs of the water and ethanol media, where the low frequencies become less prominent. The CMI component was monitored through an adequate lowpass filter, whilst the MZI was monitored by resorting to the raw transmission spectra.

### 3.3. Sensor Calibration

Prior to the sensor calibration, several mixtures of deionized water and ethanol were prepared, with ethanol concentrations that ranged from 0 wt.% to 100 wt.%, with mass fraction varying by 10 wt.%. The solutions were carefully prepared and stored to allow their stabilization.

**Figure 5:**
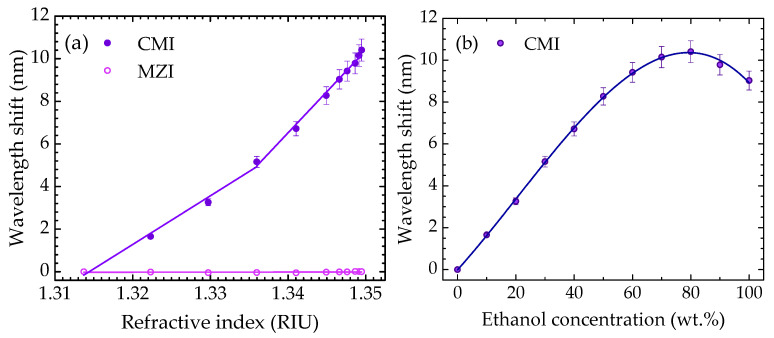
(**a**) Sensor response with refractive index variation for the MZI and CMI components. (**b**) CMI component response for the different solutions.

Figure 5a presents the CMI and MZI wavelength shifts with the liquid refractive index. The CMI was highly influenced by the variation in this measurand, while the MZI remained unchanged. This result corroborates the assumption that the CMI occurs predominantly in the outer cladding region of the HSCF, thus being influenced by the external medium changes, while the MZI occurs in the inner region of the fiber, being unaffected by these changes.

Moreover, since the CMI response to the refractive index presents a nonlinear tendency, it was necessary to delimit two regions where the response was approximately linear. In the first region, from 1.313 RIU to 1.336 RIU, a sensitivity of (229 ± 16) nm/RIU was attained, whilst for the second region, from 1.336 RIU to 1.350 RIU, a sensitivity of (387 ± 14) nm/RIU was estimated. Notice that the refractive index was estimated in the 1550 nm region, following the same procedure reported in [25].

On the other hand, Figure 5b shows the CMI response towards the ethanol concentration, which can be described by a third-order polynomial fit:

(1) $$\Delta \lambda =\left(-1.7\pm 0.1\right)\times 10^{-5}\omega^{3}+\left(11\pm 2\right)\times 10^{-4}\omega^{2}+\left(155\pm 9\right)\times 10^{-3}\omega -\left(0.02\pm 0.09\right),$$

where ω is the percentage of the mass fraction of ethanol in the mixture.

**Figure 6:**
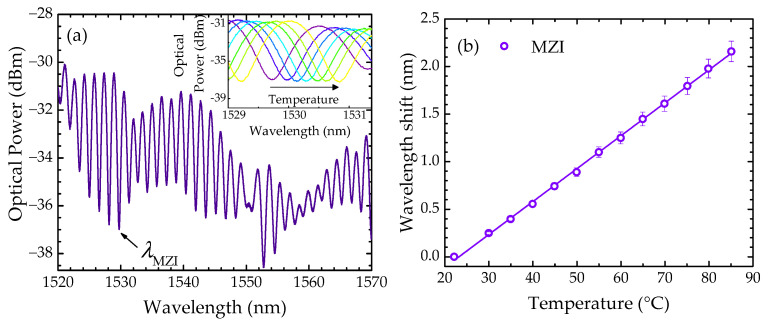
(**a**) Spectral response of the sensor in air medium at 22 °C, where the monitored MZI dip is shown. In the inset is presented the MZI dip at different temperatures (from 22 °C to 55 °C). (**b**) MZI wavelength shift dependence on temperature.

Furthermore, by resorting to the MZI component of the sensor, a temperature calibration curve was also established. The spectral response of the sensor in air medium at room temperature (~22 °C) is depicted in Figure 6a. The temperature was changed from 22 °C to 85 °C, in steps of 5 °C, using a Peltier element, which enabled a temperature reading with a resolution of 0.1 °C. The inset presents the MZI dip that was monitored during the temperature experiments.

Figure 6b shows the wavelength shift dependence on temperature; the MZI response (Δ*λ_MZI_*) can be described by:

(2) $$\Delta \lambda_{MZI}=\left(34.6\pm 0.3\right)\times 10^{-3}T-\left(0.80\pm 0.02\right),$$

where *T* is the temperature of the solution in °C and $\Delta \lambda_{MZI}$ is in nm.

## 4. Results and Discussion

### 4.1. Evaporation Analysis

**Figure 7:**
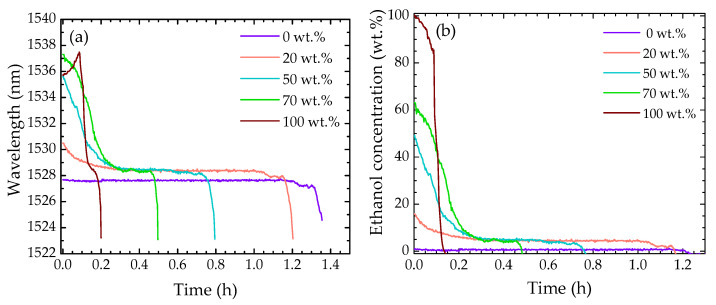
Temporal profiles of the ethanol–water mixtures with regards to (**a**) the wavelength variation and (**b**) the ethanol concentration.

The 0 wt.%, 20 wt.%, 50 wt.%, 70 wt.%, and 100 wt.% solutions were monitored to study the evaporation process. The CMI peak wavelength variation attained for each solution is presented in Figure 7a. These wavelength variations were converted into ethanol concentrations by using Equation (1). Figure 7b depicts the temporal profile of the ethanol concentration for each sample under study.

In the evaporation profiles, different stages of evaporation can be identified, according to the concentration of ethanol. For instance, during the evaporation of water (0 wt.%), the refractive index of the solution remains unaltered, leading to a constant response by the CMI. An abrupt decrease in the wavelength is observed after ~1.3 h, which corresponds to the stage of the evaporation process where the droplet had evaporated and the sensor was mainly surrounded by air.

For the solutions that contained a percentage of ethanol, namely, the 20 wt.%, 50 wt.%, and 70 wt.% solutions, a different response is observed. In a first stage, a decrease in the wavelength is verified by the decrease in the refractive index, which corresponds to the evaporation of ethanol molecules. Afterwards, the wavelength tends to stabilize at a slightly higher value than in the case of pure water. This is an indication that the remaining solution still contains ethanol molecules that did not evaporate. The reason behind this behavior is still not fully understood, though it is believed that it can be associated to the hydrophobic hydration effect [26,27] and to ethanol diffusion inside the droplet to the interface [6,28].

Regarding the 100 wt.% ethanol sample, one might expect that its evaporation would not lead to a change in the sample refractive index. However, a small increase in the wavelength is observed until ~6 min, meaning that there was a slight increase in the refractive index. This can be justified by the adsorption of water molecules from the ethanol, derived from its hygroscopicity [29]. The increase in refractive index is also justified by the curve presented in Figure 5b, where a nonlinear behavior is observable. Furthermore, the high instability of ethanol (associated with its higher vapor pressure when compared to that of water [6]), in combination with the small volume of solution, led to faster evaporation when compared with the other solutions.

Notice that, as the sensing structure is silica-based, it does not present selectivity towards the type of solutions under study. Therefore, it can be used to monitor the evaporation of other VOCs or even more complex solutions, provided that a previous calibration regarding the refractive index is performed.

### 4.2. Image Analysis

**Figure 8:**
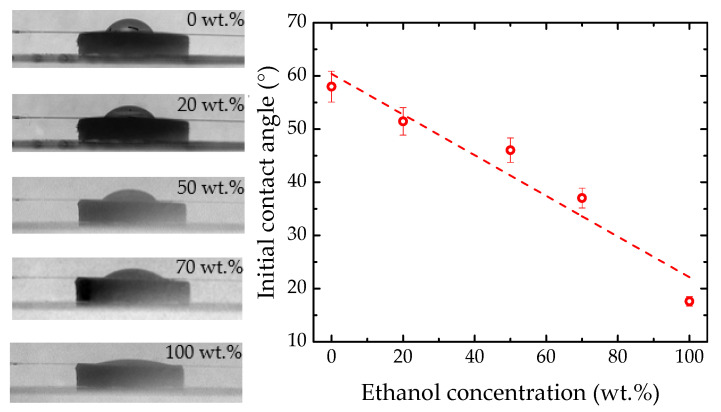
(**left**) Pictures of the droplet profile acquired of the studied ethanol solutions, for both glass and Teflon substrates. (**right**) Initial contact angles of the droplets of ethanol–water mixtures on both glass and Teflon substrates.

An image analysis of the droplet profile was also carried out to compare the results provided by both methods. Figure 8 (left) presents the micrographs of the initial droplets from the studied solutions. Notice that in all images there is a line between the substrate and the sample, which corresponds to the optical fiber sensor. For solutions with a higher ethanol concentration, the droplet will spread over the substrate. This is due to the increased wettability and decreased water surface tension with higher ethanol concentrations [5,28]. For the water sample, a spherical cap-shape is formed, a characteristic that is associated with the high roughness and wettability of the substrate [30].

Figure 8 (right) shows the dependence of the initial contact angle (*θ*) on the ethanol concentration, which was adjusted to the linear fit θ=(−0.38±0.06)ω+(60±3). This linear behavior was also reported in the literature [6,31] and provides direct information regarding not only the concentration of the solution but also the substrate properties.

**Figure 9:**
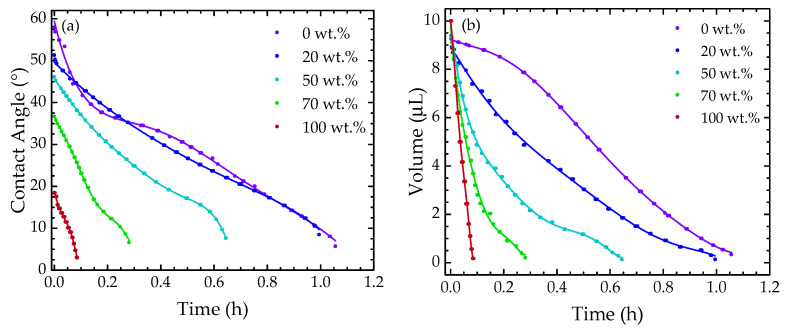

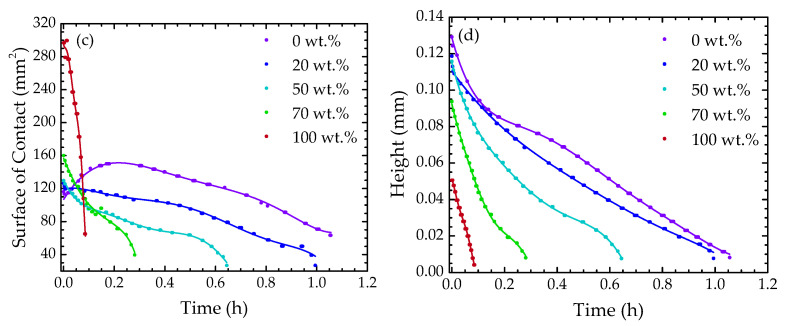
(**a**) Contact angle, (**b**) droplet volume, (**c**) surface of contact, and (**d**) height with regard to time for each mixture under study.

The images were analyzed via ImageJ software, using the “drop analysis LB- ADSA” plugin [32]. Figure 9a–d presents the evolution over time of the contact angle, volume, surface of contact, and height, respectively, of the solutions under study. During the water evaporation experiment, a slight shift in the droplet was observed through the image results. This caused the unexpected variation in the measurements between ~3 min and ~6 min. However, the optical signal was not sensitive to this change, as the whole sensor remained surrounded by solution.

Regarding the contact angle (Figure 9a), it was diminished as the ethanol concentration increased due to the decrease in the water surface tension, leading to a spreading of the droplet. For the volume profile (Figure 9b), a similar behavior was observed, being characterized by a decrease with a monotonic tendency. For the surface of contact (Figure 9c), more accentuated variation was observed for the more concentrated samples. Meanwhile, the droplet height profile (Figure 9d) decreased at an approximately constant rate. Notice that as the fiber sensor was measuring simultaneously to the image acquisition, its presence could influence the droplet profile analysis. This would be more critical if lower sample volumes were to be used. Still, notice that the results attained by the image analysis are in good agreement with those already reported in the literature [4,33].

### 4.3. Temperature Monitoring

**Figure 10:**
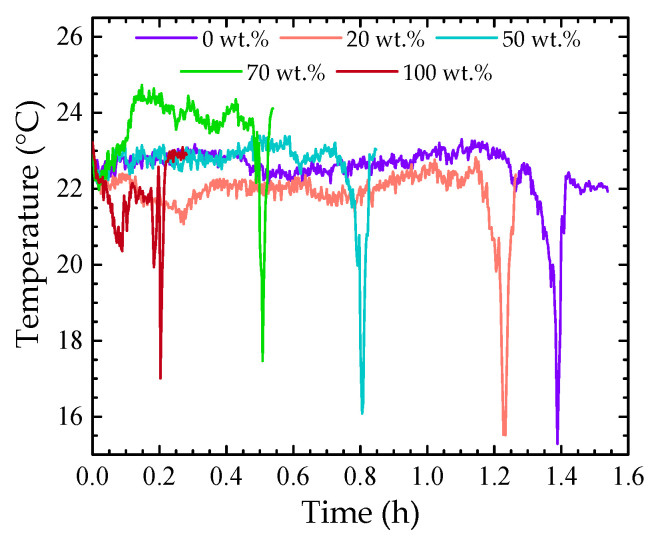
Temperature profiles of the 0 wt.%, 20 wt.%, 50 wt.%, 70 wt.%, and 100 wt.% solutions.

The sensor reported in this work can also provide valuable information regarding other parameters that may influence the monitoring of ethanol evaporation, namely, the temperature. Figure 10 shows the temperature of the system during the evaporation of the 0 wt.%, 20 wt.%, 50 wt.%, 70 wt.%, and 100 wt.% solutions, determined by using Equation (2). In the evaporation of pure substances, like water and ethanol, the temperature variation is smaller, while for the solutions containing ethanol, that variation is more noticeable and tends to increase with increasing ethanol concentration.

Notice that there is an abrupt decrease in the temperature in all the temperature profiles, forming an accentuated dip. Although the MZI response, used to track temperature variations, is sensitive to only this parameter, the measured value is not realistic. On the other hand, the CMI is sensitive to both temperature and refractive index variations. As the external medium changes from liquid to air, the CMI spectral response varies considerably (Figure 4 left), influencing the MZI response. Still, with this feature, it is possible to use the MZI not only to monitor the solution temperature fluctuations, but also to rigorously estimate the transition between media.

Furthermore, a temperature compensation technique can also be established [25], diminishing the associated errors when considering the influence of temperature in the optical sensor measurements.

## 5. Conclusions

In sum, a simple and robust HSCF sensor was proposed to monitor the evaporation of ethanol–water mixtures. This sensor was able to monitor changes in the solution’s refractive index, with a maximum sensitivity of 387 nm/RIU, and thus allowed us to achieve real-time monitoring of variation in the ethanol concentration during the evaporation process. Furthermore, an image analysis was also carried out, for comparison purposes. Finally, the sensor’s capability to monitor the sample’s temperature fluctuations was explored. This measurand may be used not only to achieve temperature compensation but also to infer some properties of the evaporation process.

The results obtained by the optical fiber sensor, in combination with the information provided by the image analysis, allow us to achieve a better understanding of evaporation monitoring, not only of ethanol–water mixtures, but also of other binary mixtures with VOCs. The image process shows the evolution of certain parameters that the optical fiber sensor is not able to detect, especially at a geometric level, and can provide an easy and quick way to initially predict the concentration of the solution. However, the sensor can detect with higher accuracy, and in real time, changes in the solution concentration and temperature as it evaporates and can provide information on the intrinsic properties of the solution under study.

## Data Availability

Not applicable.
